# Tuberculosis Under Apartheid in Cape Town, South Africa, 1950–1989

**DOI:** 10.1093/shm/hkag069

**Published:** 2026-08-20

**Authors:** René Raad, Graeme Hoddinott, Justin Dixon, Martin Gorsky

**Keywords:** tuberculosis, apartheid, South Africa, social medicine

## Abstract

This article explores the paradox that tuberculosis (TB) in the post-war period had become ‘a completely curable disease’ yet remained a leading cause of death amongst global majority populations. Cape Town, South Africa, in the apartheid era provides our case study of the structural violence that inhibited appropriate social and clinical interventions. We first review recent historical literature on TB in global health, then describe the development of TB facilities in the Cape health system, within the context of apartheid legislation. We then analyse the different aspects of apartheid which impacted upon TB medicine. Assumptions about racial difference permeated behavioural and epidemiological discussions; residential segregation and harsh controls on labour mobility confounded accurate disease surveillance and were inimical to successful drug regimens, while poverty and racial politics prevented social medicine responses in nutrition, housing, and welfare.

## Introduction

During the twentieth century, mortality from tuberculosis (TB) fell in high-income countries to the extent that the disease virtually disappeared. Its initial decline predated the emergence of the therapeutics which ultimately drove down incidence, the Bacillus Calmette-Guérin (BCG) vaccine and antibiotics. Consequently, this was attributed to economic and political changes that tackled underlying causes of the ‘social disease of the nineteenth century’, above all poor nutrition and insanitary, overcrowded housing.^1^ In other regions, including Sub-Saharan Africa and South-East Asia, this trajectory was not followed. By 2000, TB had become, paradoxically, both ‘a completely curable disease and the leading cause of young adult deaths in much of the world’.^2^ Over the preceding decades faith in biomedical cures had fluctuated, first as vaccine efficacy proved to be only partial and then as antimicrobial-resistant TB strains emerged. Yet development of new antibacterials, successfully administered in combination with others, gave grounds for continuing therapeutic optimism. The manifest failure to control TB in much of the world therefore continued to be understood in terms of social factors.

How have TB historians interested in the ‘social’ operationalised the concept? Rene and Jean Dubos’ early articulation of the disease as the ‘penalty that capitalistic society had to pay for the ruthless exploitation of labor’, still carries weight, although now reframed as ‘structural violence’.^3^ This denotes longue-durée economic factors that rendered some populations more vulnerable to illness and less able to access appropriate care. In a global perspective, it entwines with histories of colonial expropriation and exploitation, which differentiated peoples according to racial hierarchies.^4^ Upon this base were more contingent forms of the social. First, the shifting nature of the public health system set by the state, in which TB patients became subject to surveillance and management: the exercise of biopolitics. Second, the contexts, in which medical knowledge about TB was generated, were subject also to disparities of power. Finally, there was the ‘street-level’ interface at which patients and medical professionals interacted and where the behavioural determinants of drug regimens were in play.^5^

The aim of this article is to explore these social aspects of TB through a case study of Cape Town, South Africa, between c.1950, when a new mood of therapeutic optimism emerged, and the late 1980s, when HIV/AIDS triggered a major increase.^6^ Dubbed South Africa's ‘public-health enemy number one’ in the 1950s and its ‘biggest single health problem’ in the 1980s, TB remained in the 2010s its leading cause of death.^7^ By then the country would also have one of the world's highest burdens of drug-resistant TB (DR-TB), with streptomycin resistance and the need for combination therapies first recognised locally in the 1950s.^8^ The importance of the South African case lies also in the apartheid policies imposed on its population. Grounded in notions of racial difference and enforced by state brutality, these engrained systemic inequalities and distorted the health system. They cast a particularly stark light on TB's social history: an extreme example of structural violence, of healthcare within a disciplinary state, and of clinical relations and medical knowledge constrained by a racial ideology.

More generally, a close focus on a city and region offers insights unavailable at the national level. Within a limited geographical area, the full network of care facilities can be observed, and their adequacy to meeting needs considered. Specific conditions of labour, mobility, housing and nutrition can be brought into view, related to place and ‘race’. The peculiarities of epidemiological analysis founded on racial division can be explored, and causes of reporting errors explained. Finally, the growing awareness of antimicrobial resistance (AMR) can be tracked and its implications for local policy explored.

In what follows, we look first at the global and national historiography of TB, charting a consensus that reliance on biomedical over social solutions led to policy failure. Next is contextual discussion of how the Cape's public health structures were organised within the apartheid state. We then present local epidemiological data on TB incidence, demonstrating how apartheid legislation distorted accurate reporting. Our central section sets out the main public policy trends over the four decades, noting both developments in chemotherapy, the hope, then disillusion, attending the BCG vaccine, and the practical issues surrounding diagnostics. Our analysis attends particularly to how segregation and discrimination interacted with policy initiatives. Finally, we discuss the scope for a social medicine approach, viewed principally from the vantage of nutrition.

## Studies in failure

Recent global histories of TB have established some clear explanatory contours. Overall the tone is one of resignation, for there is no progressive narrative of ‘conquest’ to relate.^9^ Rather, there were phases of optimism, misplaced confidence, and then ‘languished hopes’ as new panaceas came and went.^10^ Most agree the mid-century was a moment of insight when theories of racial susceptibility to TB infection, sometimes conflated with ‘virgin soil’ accounts of vulnerable populations, were jettisoned.^11^ Instead was acknowledgment of the primacy of structural determinants, and hence that socio-economic improvement coupled with universal health coverage was the remedy.^12^ This chimed well with the era's internationalist discourse of social medicine.^13^ In practice, though, policymakers turned to the BCG vaccine as the optimal strategy for poor settings.^14^ Interpretation varies on why social interventions were abandoned. Brimnes emphasises the WHO's support for vertical and technical eradication programmes and India's influential embrace of BCG as a symbol of modernity.^15^ McMillen stresses the blend of desperation, realism and economic calculus that motivated the vaccine's proponents, coupled with misplaced trust in the promise of early trials.^16^

The rise of drug treatments is understood in terms of disillusionment with BCG's failures and faith in the modernity of Western medicines. Mass-produced, standardised and validated, their legitimacy as global tools was grounded in late-colonial settings.^17^ Gradmann notes the ‘exaggerated trust’ placed in chemotherapies, while McMillen emphasises the pragmatism of supportive doctors determined to save lives.^18^ A chronology crudely runs from the 1950s with streptomycin, isoniazid (INH), and para-aminosalicylic acid (PAS), followed by take-up of thiacetazone and ethambutol, then in the 1960s, with awareness growing of the need for combination therapies, the arrival of rifampicin and expansion through ambulatory, not hospital, care. AMR, despite its current salience, is traced to laboratory researchers from the 1900s, leading scientists since the 1940s, and periodically to TB chemotherapies.^19^

In the late twentieth century, Gradmann finds the WHO's adoption of ‘DOTS’ programmes (‘directly observed therapy’, to ensure patients completed their medication) as a harbinger of 1990s ‘global health’, driven by metrics and cost-effectiveness. McMillen leans more towards continuity with earlier practices.^20^ Historians also problematise the notion of patient ‘noncompliance’ with drug regimes, seeing this as a social construct that blames TB sufferers. Rather, in Seeberg's words, an ‘iatrogenic epidemic’ was fuelled by imposing strict chemotherapeutics on fragile health systems.^21^

South Africa already has a distinct place in this literature. For example, Durban TB specialist Basil Dormer is credited with dislodging theories of racial predisposition in favour of social factors.^22^ However, most texts are also written in a register of ‘gloom and pessimism’.^23^ In the early 1980s, historical articles by practitioners pondered ‘where have we gone wrong?’ with South African TB control, ignoring apartheid and blaming poor knowledge, underfunding and insufficient testing.^24^ It was Packard's 1989 focus on mining conditions in the Rand which showed how apartheid labour policies exacerbated TB's spread and intensified transmission.^25^ He also debunked the much-touted parabolic line graph of South African TB notifications, whose n-curve rose to a late-1950s plateau, then fell sharply. Once a cause for modest satisfaction, this was now revealed as an artefact of undercounting due to forced removals.^26^ The end of the regime ushered in more candid histories. McCullough revealed how mining companies ‘manufactured ignorance’ of long-term TB risks to avoid paying later compensation to repatriated migrant workers.^27^ Van Rensburg et al. delineated the specific contributions of apartheid to worsening the situation: poor living conditions and inadequate social security; fragmented health services; weak public hospital provision; and enforced labour mobility due to residential segregation.^28^ South Africa's TB histories expounded a ‘bleak prospect’.^29^

What can a study of Cape Town and its surroundings add? Scholars have noted that prior concentration on mining areas conveyed only a partial perspective.^30^ The Cape region's economy was rooted in diverse sectors, with a distinct labour market attractive to in-migrants. Although it developed one of the country's most severe TB burdens, it was relatively advantaged with respect to TB strategy, having ‘led the field in promoting inter-colonial investigations’.^31^ Its health system was comparatively well-functioning and resourced, serving as a ‘testing ground’ for novel interventions, pharmaceuticals and regimens.^32^ Another distinguishing feature was the high proportion (∼50% of Cape Province, 1948–1989) racially classified as ‘Coloured’. A portmanteau term for mixed ethnic backgrounds, such as Khoi, San, White, Black, Malay, Griqua, and sometimes Indian or Asian, ‘Coloured’ embraced both diverse heritages and a collective cultural identity, remaining in use as a self-description today.^33^ Examining the differential experiences of ‘Coloured’ persons further illuminates intersections of public health strategies with structural factors.

## Methods and terminologies

Our findings are based on the synthesis of documents relevant to the science, politics, and epidemiology of TB in our period. This selection criterion regrettably reflects the exclusionary apartheid state, for the voices are almost entirely those of White officials and doctors. Primary sources were collected from the Western Cape Archives and Records Service, the Cape Town Records Centre, and the National Library of South Africa (Cape Town Campus). Prime among official documents are the annual reports of the Medical Officer of Health (MOH) for Cape Town, a mine of epidemiological and policy detail: as public-facing texts, they were naturally cautious, although sometimes included social and political criticism. Institutional records, such as minutes and correspondence of government and local charities, were also used, and various newspapers and journals, notably the *South African Medical Journal* (*SAMJ*). The latter was affiliated with the Medical Association of South Africa, whose complicity with apartheid later provoked international condemnation, with the *SAMJ* similarly criticised for suppressing critical views.^34^

Throughout this article, we engage with contemporary categories such as ‘non-European’, ‘Bantu’, ‘Black’, and ‘Asian’ and ‘Coloured’, which run through the documentary sources. ‘Race’ under apartheid was a socially and politically manufactured classificatory system divorced from objective reality.^35^ Its classifications were state-imposed descriptors that institutionalised racial hierarchies and regulated access to resources, including healthcare. Naturalised and enforced under apartheid-era public health policies, we necessarily repeat such terminology here, mindful of the tensions involved in their usage.

These are particularly acute with TB statistics, where racial classifications structured how data were recorded, interpreted and used in decision-making. As Stoler and Burton argue, colonial archives are not passive repositories of information but instruments of governance that reinforce racial hierarchies and exclusion.^36^ Within the apartheid state, the selective collection and classification of TB data functioned as part of a broader strategy to manage African health in ways that served White economic and political interests. This consideration shapes our documentary analysis.

## TB in the Cape health system

This section sets out the context for the study, establishing the political and administrative structure of the Cape health system and showing how the network of hospitals and clinics which managed TB were stratified by racial categories. In Cape Town, the convergence of rapid urbanisation, industrialisation, and poor housing intensified conditions conducive to TB's spread, and local services emerged slowly. Spatial and occupational segregation marked the early phase of institutional responses, and after a brief post-war moment when more universalist strategies were propounded, this was consolidated under the apartheid regime.

First, how did the disease emerge here? Although genomic analysis suggests an African origin of *Mycobacterium tuberculosis*, it was probably at low levels of endemicity before the mid-nineteenth century.^37^ This changed when consumptive migrants tempted by the salutogenic climate began settling in the Karoo region.^38^ Discovery of deposits of diamonds (1867) and gold (1886) triggered an influx of workers. Industrialised mining transformed South Africa's predominantly agrarian economy, and TB spread amongst migrant workers and their rural-dwelling families.^39^ In 1910, the Union of South Africa was created, joining the previously rivalrous British colonies and Boer republics into a unitary state. The formation of the union was significant for TB when, accelerated by the crisis of the Spanish flu, it passed a first Public Health Act (1919). This empowered local government to demolish slums and plan housing, inaugurating the residential segregation that would marginalise the vast majority of South Africans and entrench urban privileges for Whites.^40^ It also established three tiers of governmental responsibility for public health, national, provincial, and municipal, which for TB would lead to tension over resources and policy.

Institutional provision developed gradually. Available interventions were prolonged hospitalisation, rest, dietary supplements like cod-liver oil, and, in severe cases, invasive surgeries.^41^ Notification became compulsory in 1904, and a TB clinic was held at the City Hospital where patients might be admitted or sent for isolation to Nelspoort Sanatorium, 370 miles distant in the Karoo.^42^ Further TB clinics opened at Newmarket Street (1929), newly incorporated Wynberg (1935) and Windermere (1943), with affiliated health visitors undertaking home visits.^43^ Langa Native Hospital was the other main provider for TB, but the Cape struggled with severe bed and staff shortages.^44^ Planned as a model town of barracks and small houses for ‘natives’ from 1923, at mid-century Langa also had a TB clinic and maternal and child welfare crèche.^45^ In 1937, a proposed 200-bed expansion for Cape TB inpatients was vetoed in favour of a cheaper clinic at Chapel Street, with X-ray and laboratory facilities.^46^

By 1948, Cape Town's economic vitality hinged on its role as a global trade nexus between Europe and the East Indies, and it attracted much migrant labour. Hitherto, a comparatively mixed city, it would now be shaped by the apartheid state constructed by the newly elected National Party (NP). Its Population Registration Act (1950) classified individuals into racial categories, and from 1952 Black people were obliged to carry a ‘pass book’ when outside their ‘homeland’. This enabled the policing of African labour mobility, while the Group Areas Act (1950) further entrenched racial segregation by place.^47^ Drastic curtailment of Coloured people's voting rights in 1956 consolidated White supremacy.^48^ Designated areas for specific races emerged, with ‘non-Europeans’ forcibly removed from urban centres. Major racial designations were Athlone in the Cape Flats for Coloured people and the Black townships Langa, Nyanga, and Gugulethu.^49^ This enforced spatial separation would determine economic opportunity, settlement, and health service provision.

The mid-1940s saw an attempt by Henry Gluckman, Minister of Health 1945–1948, to centralise healthcare and create more comprehensive social support.^50^ Gluckman's 1944 Report, *à la* Beveridge, favoured expanding health service provision and took a social medicine perspective, acknowledging environmental determinants.^51^ He proposed a universal national health service to include adequate mental, dental, nursing and hospital services and extensive primary healthcare.^52^ With respect to TB, he implicated poor housing and malnutrition as causal, particularly for ‘Natives’, and urged a trebling of expenditure to fund hospital building, ideally in ‘pleasant rural surroundings’, for effective isolation.^53^ In Gluckman's wake, medical allies openly advocated against ‘discriminatory legislation’ like colour bars for labour to benefit ‘non-Europeans’ facing social diseases like TB.^54^ However, this vision fell victim to apartheid, and such frank rhetoric became uncommon.^55^

What was post-war Cape Town's position in the tripartite division of authority? The national Department of Health established health policy, regulations, and standards of care aligned with the regime's objectives and provided funding for infrastructure and treatment. Within this framework, provincial health departments retained some independence, developing policies tailored to their populations.^56^ In Cape Town, municipal health governance was split between the city council and the Cape Hospital Board, which oversaw the larger region. The result was devolved arrangements heavily dependent on central grants: Nelspoort Sanatorium, for instance, was funded by the government but Cape Town's MOH sat on its advisory board and allocated patients.^57^

The Cape's segregated hospital services developed throughout our period. At its start these included Brooklyn Chest Hospital (originally Rentzkies Farm Hospital) for ‘non-European males’; City Hospital, with a mixed ‘European’:‘non-European’ TB beds ratio (64:84), Nelspoort Sanatorium (37:26); the Sunshine Home for Children (60:42); and Langa Native Hospital, exclusively for Black citizens.^58^ Despite this, pressure for beds intensified after the Chapel Street clinic began mass radiography in 1948, initially screening large numbers sent from factories and workplaces.^59^ Increased case-finding exacerbated bed shortages: by 1955, only 14% of new diagnoses could be admitted to hospital. Initial relief came in 1950 when the Dr A.J. Stals Memorial Sanatorium with c. 50 beds for ‘non-Whites’ opened on a government site in Westlake.^60^ Close by, the D.P. Marais Tuberculosis Centre opened in 1957 with 100 beds for ‘Coloured males’.^61^ This was founded by the South African National Tuberculosis Association (SANTA), a voluntary organisation established in the 1940s, whose fundraising provided 28 TB settlements nationwide by 1958, with 5,500 new beds.^62^ Typifying apartheid's mixed economy, the Marais Centre's buildings were provided by SANTA, with inpatient funding split between the Cape City and Divisional Councils, of which seven-eighths was recoverable from central government.^63^

With hospital accommodation for ‘non-Europeans’ always insufficient, ambulatory TB care was introduced by necessity, with patients receiving chemotherapies at home, work, or in clinics.^64^ Further ‘non-White’ clinics giving TB treatment opened as the city sprawled, including Athlone (1954), Nyanga West (1959), and Gugulethu (1964); in 1967, Langa received an X-ray machine through which Black migrant arrivals were channelled, and Nyanga West had a mobile X-ray van.^65^ A mid-1970s reorganisation into new health areas with community polyclinics led to further expansion, with 14 TB clinics by 1978, including new facilities in the suburbs Retreat and Westridge; shortly after, Langa's new polyclinic replaced the now ‘totally inadequate’ hospital unit.^66^ Concurrently, though, TB hospital beds were lost, with reductions at the Stals Sanatorium, now an institution for ‘Coloured defectives’, the Marais Centre and City Hospital. By 1984, the MOH was strenuously deprecating the ‘grave error’ of closing 1,000 TB beds ‘for financial reasons’.^67^

The intensifying bed crisis of the 1980s also reflected the advance of private medical care. The central government's National Health Services Facilities Plan in 1980 signalled the end of public funding growth for hospital beds, nominally to promote health centres instead.^68^ However, real per capita health expenditure then remained flat, as South Africa's financial crisis worsened amidst political instability.^69^ Meanwhile, the NP's neoliberal turn encouraged private medicine; healthcare was now an individual utility, not a public good, and insurance would foster ‘self-reliance’.^70^ A two-tier system emerged, in which, by the mid-1980s, 70% of the White population was insured, but only 7% of non-Whites (mostly trade unionists), with 26% of beds and over half the nation's doctors in private hospitals.^71^ In Cape Town, this did not benefit TB programmes, for private general hospitals concentrated on surgical and ‘minor short-stay’ cases.^72^ Only the for-profit Airemount Nursing Home took TB referrals from the City and Divisional Councils, catering to ‘Europeans’.^73^

In sum, our period began with a TB hospital bed crisis, which was gradually ameliorated by SANTA and central government. Screening, coupled with ambulatory care, seemed to promise reduced need for hospital provision, and clinics proliferated to run the programmes. However, these proved insufficient, and the apartheid era closed with another crisis of capacity, born of consistent under-resourcing. To begin our more detailed examination of that failure, we next consider the statistical record.

## 'Tuberculosis occurs where it is looked for’: historical epidemiology, 1950–1989

In making policy it was of obvious importance to quantify the scale of TB mortality and morbidity and to plot their variations over time. Unfortunately, the gathering and presenting of statistics in our period were so vitiated by strictures of apartheid that they cannot be viewed as valid indicators of population health. To an extent, this is true of all TB incidence metrics, for while mortality records are usually robust, disease notification is notoriously unreliable.^74^ Even in homogeneous, wealthy societies, it can fail through patient concealment or medical reporting omissions. In South Africa, though, the added distortions of spatial segregation and labour mobility mean the data cannot be read as unproblematic representations of disease ecology. They can nonetheless disclose useful evidence.

First, they take us into the mind-set of South African governance, in which experience could be conceived only through racial categories. Figure 1 presents the annual population as recorded in the MOH reports using the four main classifications applied to TB data. The Black count initially excluded dwellers of Langa and Nyanga West, whose ‘rapidly changing migratory population’ was deemed too uncertain.^75^ The ruptures in trend, 1961 and 1985, are simply what was reported and presumably relate either to the exclusion of in-migrants or to boundary changes. These statistics present ‘Cape Town’ growing from 415,000 to 1,200,000 inhabitants by 1989, although the first post-apartheid census (1996) recorded 2,563,000 persons in the whole metropolitan area.^76^ They are then representations that normalise geographical and racial segregation.

**Figure 1.**
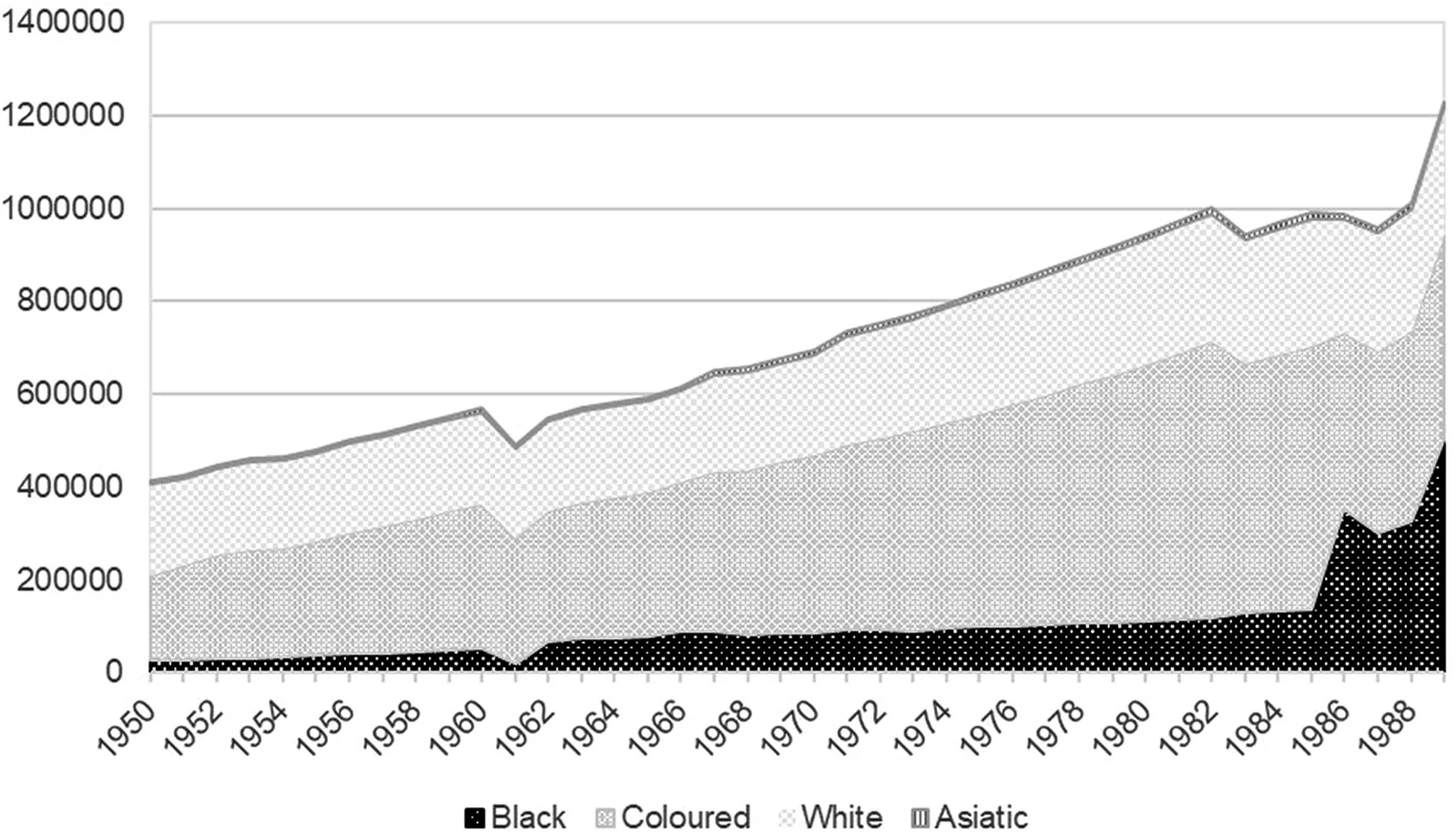
Population of Cape Town, by ‘race’ categories (1950–1989).

Second, despite incompleteness, they do capture *some* patterns. Figure 2 shows Cape Town's mortality rate from pulmonary TB according to the racial classifications. These figures are not a straightforward gauge of acute TB trends. The MOH frankly acknowledged the under-reporting of Black people's deaths due to the ‘egress of Natives who … have returned to the country to die’, so that any downward ‘swing in Cape Town is lost in the rondevals of the Transkei’.^77^ Similarly in 1989, inaccuracy was blamed on ‘many administrative problems with obtaining Black mortality data’.^78^ That said, the marked decline for the White and Coloured groups in the early 1950s plausibly reflects the new regime of testing and antibiotics and explains early post-war optimism. While the exact degree of Black undercounting is unknowable, the data also show that although overall mortality dropped to virtually zero for Whites, the ‘non-White’ populations remained excessively affected.

**Figure 2.**
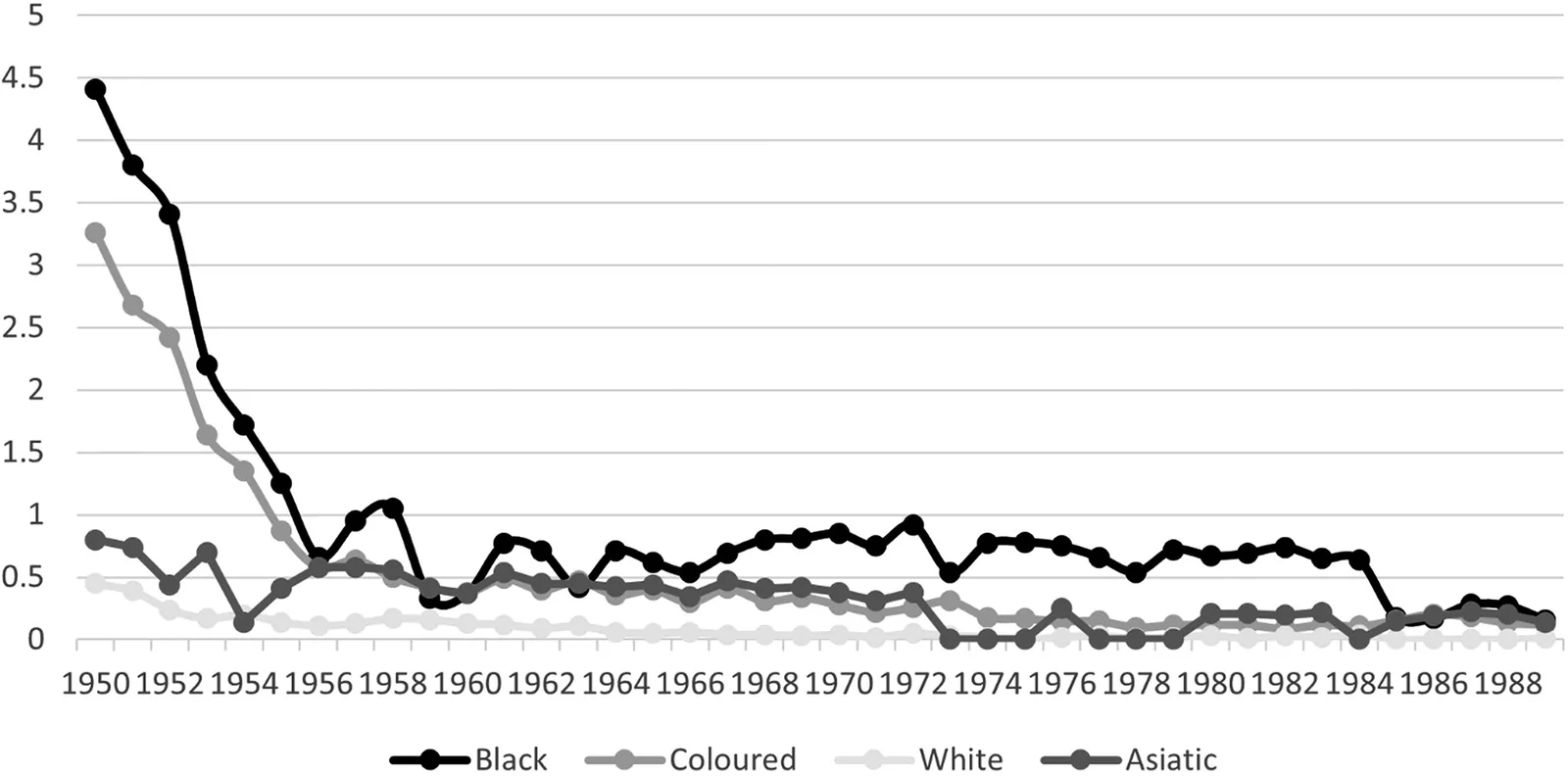
Mortality rate (per 1,000) for pulmonary TB, by ‘race’ categories (1950–1989), in Cape Town.

Figure 3 presents the notification rate of pulmonary tuberculosis in Cape Town, 1950–1989, disaggregated by race. Treated by the MOH as a proxy for incidence, this was the annual total of new cases diagnosed and reported as a proportion of 1,000 population.^79^ Probably, the time series for Whites captures incidence reasonably accurately, the gradual decline almost to zero founded on better living standards and access to services. Probably too, the decline in Coloured notifications is indicative of the substantial gap with Whites narrowing until 1973, from when they increased. Black notification rates were far higher through most of the sequence, although it is unlikely this series bears much relationship to actual incidence, with peculiarities like the troughs of 1957–1961 and the fall from 1985.

**Figure 3.**
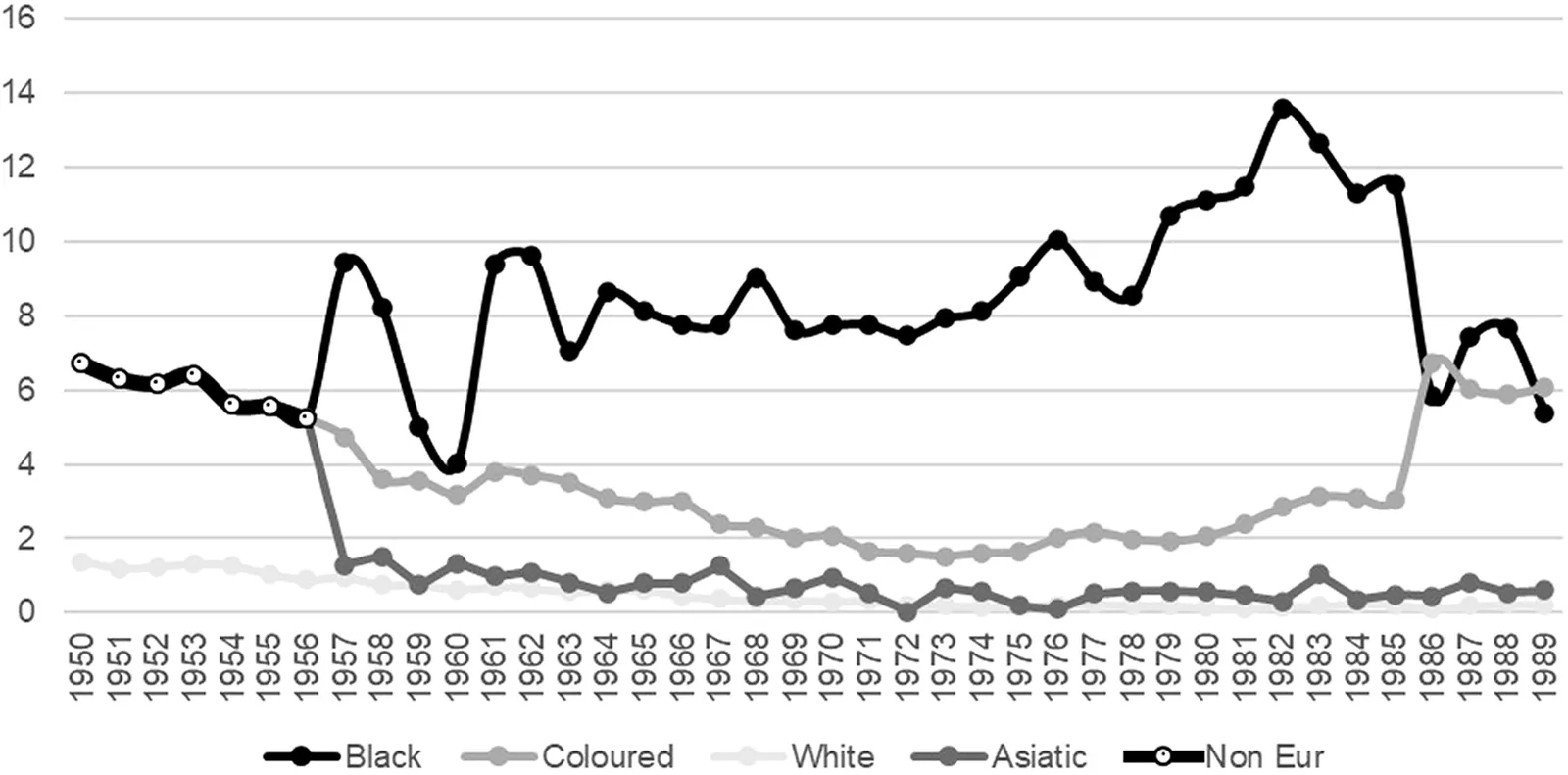
Notification rate (per 1,000) for pulmonary TB, by ‘race’ categories (1950–1989), in Cape Town.

In fact, the fragility of the underlying data was regularly stressed by the authorities. Reflecting on the scale of notifications in the first year of mass radiography, TB officer, W.L. Hoole, noted ‘the uneasy truth that tuberculosis is found in Cape Town wherever it is searched for’.^80^ This observation, that TB ‘occurs where it is looked for’, repeatedly appears in the sources, helpfully illuminating the graphs’ true nature.^81^

Thus, high rates in the 1950s were attributed to the introduction of mass radiography, then to the clinics’ increasing reach, with the first ‘upsurge’ of Black cases related to the ‘intensified search’ at Langa's and Nyanga West's.^82^ The subsequent 1957–1961 trough was explained by ‘staff difficulties and shortages’ and ‘civic disturbances’ in the townships.^83^ Expanding clinic numbers in the 1960s similarly caused a ‘largely artificial’ notification rise at Gugulethu, while Coloured decline was partly due to enforced removals.^84^ From the late 1960s, high Black rates were related to the new X-ray machine at Langa ‘through which all Bantu work seekers are now channelled’.^85^ The mid-1970s dip was attributed to a recessionary fall in in-migration and a fire in the X-ray building.^86^ Meanwhile, Coloured patients with Langa or Gugulethu addresses were excluded from statistical calculation, thereby understating the late-1970s rise.^87^ By the 1980s, MOH figures came with standard warnings about unreliability, while the late-1980s fall was due to changing population estimates.^88^ Cape Town researchers, comparing notification rates with prevalence surveys, concluded the former were ‘the tip of the iceberg’, with true incidence probably ‘two to three times more’.^89^

In sum, the available statistics probably give a fair picture of improving White mortality and morbidity trends, and perhaps a rough impression of Coloured people's experience, with apparent improvement later unravelling. They are, though, unusable for gauging the impact of TB amongst Black people. Under-reporting, demographic shifts and the socio-political complexities of data collection undermined reliability, as did the inadequacies in health infrastructure.^90^ Above all, these statistics demonstrate the racial frameworks through which TB was viewed and which shaped policy-making. It is this to which we now turn.

## Failures of prevention and cure: BCG campaigns in the Cape, 1957–1987

Histories of post-war global TB responses typically foreground the role of BCG campaigns.^91^ In the Cape, however, these were less prominent. South Africa was initially cautious, with Dormer arguing vaccines were inappropriate for Whites or tuberculin-positive individuals and for ‘Bantu’ only if alongside social interventions.^92^ The *SAMJ* led a 1953 issue with a sceptical article by American TB expert Arthur Myers warning BCG ‘remains an experiment … one of the most controversial … in the history of medicine’.^93^ However, results of the British Medical Research Council (MRC) vaccine trial showing an 82% reduction in incidence were reported in 1956, and next year Cape Divisional Council ran a pilot scheme for children with a ‘small supply’ of Glaxo vaccine. Then, in 1959, BCG was introduced for infants in Cape Town's clinics and maternity homes.^94^ By the 1960s, this was routine, with state-supplied, freeze-dried vaccine administered to all newborns, either in hospital or by health visitors.^95^ Administratively the Child Welfare Branch ran the programme, alongside immunisation against diphtheria, whooping cough and polio. There was limited integration with broader TB policy, beyond inoculating ‘contact children’.^96^

Confidence in BCG rose in the 1960s when research demonstrated its safety with positive reactors and its utility for prevention and diagnosis.^97^ As awareness grew that non-completion of drug treatment led to resistant TB strains, immunisation assumed greater urgency.^98^ Government made vaccination compulsory within 6 months of birth in 1973, unless parents objected in writing, and Cape Town schoolchildren were periodically revaccinated due to reliability concerns.^99^ Meanwhile, optimism waned with discouraging local research. In 1967, a survey of schoolchildren vaccinated 2 years earlier found an average of only 52% tuberculin conversion.^100^ A 1977 Johannesburg study discovered 23% of a vaccinated group later developed TB, prompting worries about vaccine quality and counter-effects of malnutrition and multimordity.^101^ It also became clear that routine immunisation had not reduced notification rates for extrapulmonary TB, which instead had remained stable: hence, ‘notified incidence … is largely independent of BCG vaccination’.^102^ Overall the protection rate was now calculated to be about 60%, and BCG's contribution to TB decline was only ‘minimal’.^103^ In 1987, compulsion was withdrawn, and Cape TB experts expressed increasing diffidence as to BCG's value.^104^

## Failures of prevention and cure: ambulatory treatment and the ‘short-term campaign’, 1950s and 1960s

Instead of faith in prevention then, the Cape's policy focus was on detection and the ‘miracle’ of chemotherapy.^105^ However, in the first decades of the apartheid state, its benefits were not equally shared. Mass radiography since 1948 had turned up a large reservoir of hitherto hidden cases, yet drug supplies were initially insufficient for ‘all in clinical need of them’.^106^ Given that this period was when White TB notifications and mortality fell decisively, we can assume that it was ‘Europeans’ who had priority access to antimicrobials. They also benefitted from segregated services in hospitals and sanatoria, while White-only clinical sessions were held at Chapel Street and Wynberg (fig. 4). The TB officer frankly acknowledged racial advantage, attributing the divergent outcomes to ‘the contrasting facilities for the two groups’.^107^

**Figure 4.**
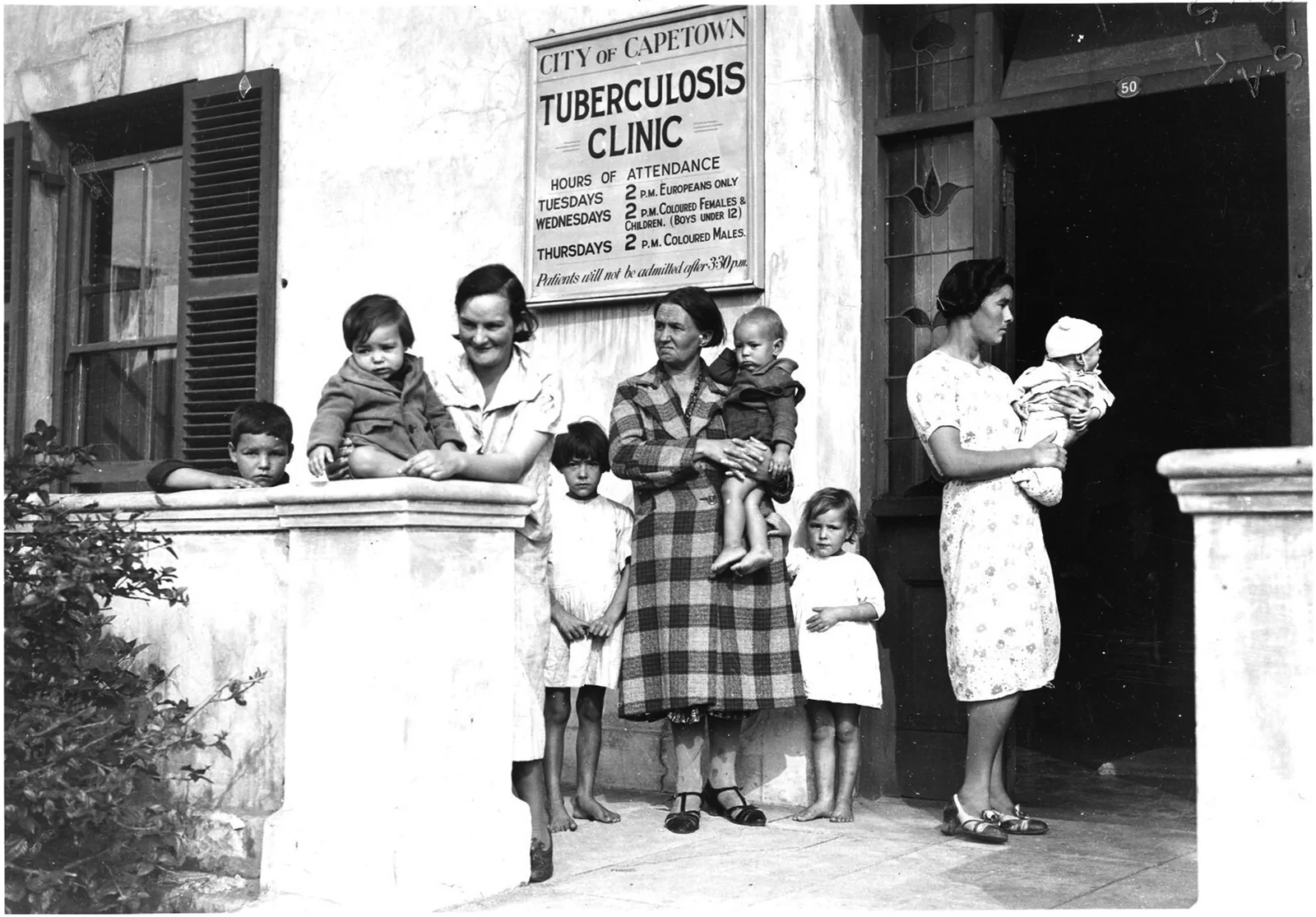
‘City of Cape Town, Tuberculosis Clinic’. Source: Cape Times Collection, by permission National Library of South Africa

What about ‘non-Europeans’? Coinciding with the arrival of chemotherapies, the Cape's post-war crisis of hospital accommodation was exacerbated by reluctance to endorse racially mixed staffing.^108^ Scarcity prompted the beginnings in 1954 of ‘domiciliary treatment’. Funded to the tune of 87.5% by the national government, this involved two nurses visiting patients’ homes and giving intramuscular injections of streptomycin, with PAS and INH tablets.^109^ According to the *Cape Argus*, this was the first such policy anywhere in the world which allowed patients under treatment to remain at work.^110^ The hope was that by shortening waiting lists, community exposure to infectious people would also be reduced. Probably the opposite occurred, for poor housing and incomplete regimens maintained patients in the home and workplace while still infectious.^111^ Official reports condemned the contributory role of social conditions under apartheid, albeit cloaked in euphemisms like ‘civic conscience’.^112^ Overcrowded living conditions, the ‘exposure and fatigue’ of factory labour, limited facilities and the reluctance of ‘Native men’ with TB to present until too late were all observed.^113^

The later 1950s saw further hardening of biopolitical apartheid, from the ‘petty’, such as the Immorality Act, which banned White/non-White sexual relations, to the Grand Apartheid programme of Hendrik Verwoerd, Prime Minister from 1958. Premised on ‘separate development’, this entrenched White supremacy, with the rigorous application of the pass laws to regulate Black people's access to urban areas. Cape TB policy also embraced heightened surveillance and control. In 1957, the Divisional Council's ‘short-term campaign’ began, signalling more aggressive case-finding efforts through the mobile X-ray units that began traversing the peninsula, then utilising drug therapies, new SANTA settlements and government aid.^114^ The scale of activity was considerable. The five city clinics (Chapel Street, Wynberg, Windermere, Athlone, and Langa) conducted 11,432 primary consultations in 1958 for the newly notified, suspects and contacts, of which 82% were ‘non-European’. They also provided 46,405 streptomycin injections for ambulatory patients and carried out 18,179 screenings. A further 18,219 domiciliary injections were given by health visitors, who made 38,555 home visits.^115^ The hope then was to reach hitherto ‘difficult to control’ patients who had refused hospital treatment in clinics or at home.^116^

However, the enmeshing of TB control measures with apartheid's restrictions was problematic for both patients and officials. Labour policy and enforced residential segregation shaped Black people's health behaviours, obfuscating management data, for there was no way to detect those ‘ill and infectious’ in-migrants to Langa who then obtained work or work-seeking permits in the city. Such cases became the ‘neglectful’ and ‘fatalistic’ who either presented too late or returned to die in the homelands ‘without full awareness of their plight’, thereby fostering the rural disease reservoir.^117^ Then there was the ‘flight of the workless and itinerants’ who evaded employment screenings, and the ‘illegally resident’ who ‘decamped on notification’ and became untraceable.^118^ These concerns over ‘Bantu work seekers’ carrying TB into the city prompted a screening check in 1966, which revealed 2.25% of new entrants were afflicted. To mitigate the health and financial burden this imposed, programmes for early diagnosis, mandatory screening, and limitation of settlement status were instituted.^119^ Then came the new mass X-ray unit at Langa Hospital, to screen ‘apparently fit and well Bantu’ arriving from the homelands.^120^ What ought to have been a benign preventive initiative was therefore entangled in Grand Apartheid's controls over movement, fostering non-co-operation and resentment.

The low pay of Black workers also disrupted surveillance and treatment. Voluntary attendance for screenings fell off, attributed to disruptions to wages and transport difficulties.^121^ Male breadwinners failed to seek TB clinic treatment due to the financial consequences of work absenteeism, while women were loath to pass childcare to ‘mothers, mothers in law, grandmothers and aunts etc [who] are already overburdened’. Nor were there sufficient institutional places for children: ‘So, a TB mother hugs her baby to her breast with … one or more little mites clinging to her skirt and refuses admission to hospital or SANTA’.^122^ Yet as awareness grew from the late 1950s that drug treatment failures were creating a ‘Survival without Recovery’ pool, the blame fell on patients for ‘late discovery … and the misapplication of therapy, whereby the victims, too often from individual default, are carried over to the chronic and usually infectious stage’.^123^

This was the context in which understandings of ‘non-compliance’ emerged and the consequent problem of drug resistance. Dormer warned in 1960 that irregular treatment led to ‘inability to cure’, noting the indications of resistance in sputum tests and detailing a ‘combination of drugs’ for therapeutic regimes.^124^ In an early local marker of DR-TB, Cape Town's clinics recorded 1,587 cases requiring additional INH and PAS treatment in 1959 through to 1,410 in 1963 when statistics ceased.^125^ Most alarmingly, a retrospective study of 449 patients (excluding Black males) discharged from the City Hospital in 1962–1964, revealed that 48.2% had acquired resistance either to streptomycin or to INH and that 8.2% had primary drug resistance. Patients described regimes that were intermittent, inadequate, or too brief, and the researchers duly recommended a 6-month triple drug combination regime, followed by INH and PAS as ongoing outpatients.^126^ The beginnings of TB's causal role in AMR were therefore discernible during the ‘short-term campaign’, as was its aggravation by apartheid.

## Failures of prevention and cure: a ‘new approach’, pharmaceutical emphasis and ambulatory care, 1972–1979

The 1970s saw apartheid continue its repressive course, although now facing more concerted opposition. The Bantu Homelands Citizenship Act further diminished civil rights, allotting all Black citizens to one of the bantustans, while in Cape Town the destruction and removals of the diverse District Six were the grossest example of spatial segregation. Attempts to enforce school teaching in Afrikaans triggered the Soweto Uprising in 1976, but the protest was brutally suppressed. 1977's murder by police of the Black Consciousness leader Stephen Biko galvanised the anti-apartheid movement at home and internationally. In national TB policy, a nominally progressive ‘New Approach’ was announced in 1972, which emphasised community medicine.^127^ Bouyed by research which found hospital regimes no more effective than ambulatory, it promoted outpatient and clinic treatment and ended the construction of new TB hospitals.^128^ However, apartheid's labour and population policies were inimical to the success of these programmes.

At the decade's start, Cape officials felt bed capacity was sufficient for all in need of hospitalisation and that ambulatory care, principally with streptomycin, could manage the rest.^129^ BCG coverage also ramped up in schools following compulsion, and more clinics opened.^130^ However, gains to surveillance from the Langa mass X-ray were increasingly offset by loss of employers’ support and Chapel St's diminished role following the demolition of District Six.^131^ Other apartheid policies impeded control. Unrecorded births to ‘illegally present’ mothers and the constant renewal of the infectious pool by new arrivals were repeatedly noted by the TB officer, who envisaged no improvement without the ‘abolition of the migrant labour system’.^132^ Political tensions manifested even more directly in 1976, when the Gugulethu clinic was ‘razed by fire’ and ‘minor damage was occasioned’ to others.^133^ This was the solidaristic rioting which followed the Soweto students’ revolt. We do not know whether this was intentional destruction of symbols of the racist state, like that meted out to post office, school, and university buildings, or akin to the more wanton attacks on shops and shebeens.^134^ Clearly, though, political resentment at apartheid cut across public health goals.

The consistently high rates of notification for Black and Coloured peoples also reflected problems of managing drug treatment. The Cape MOH reports began monitoring this statistically, tabulating proportions of primary notification cases that subsequently failed to attend clinics, disaggregating those refusing treatment or becoming untraceable. These amounted to only about 2.4% on average in 1975 (mean 62 of 2,618 cases) and 4.6% in 1976–1980 (mean 118 of 2,627).^135^ This, though, was the overtly quantifiable risk pool, and numbers that relapsed or acquired resistance through incomplete, inadequate, or unsupervised drug regimens went unknown.^136^ Research into resistance had moved from Cape Town to Pretoria's MRC Tuberculosis Research Institute (TBRI), which, from 1965, conducted regular national surveys based on laboratory analysis of sputum samples from selected hospitals. This showed Black people's resistance to streptomycin at 33.8% (1965–1970), falling to 10.6% (1971–1979), and to INH 28.8%, falling to 10.6%. The decline in resistance based on hospital regimes probably provided false security about risks of ambulatory treatment, for primary resistance to INH, a fair indication of its presence in the community, was on the rise: 14% (1965–1970), 16% (1971–1974), and 18% (1975–1978). Moreover, ethnic differentiation revealed the Cape-based Xhosa to have the highest level of INH resistance, 34.7%, in 1965–1979.^137^

From 1973, the newly available drug rifampicin raised global hopes for the potential of short-course chemotherapy, transforming standard treatment regimens to 6 months.^138^ However, its significance here remained negligible. Cape Town patients continued to receive principally streptomycin cures, with INH prophylaxis for those with latent TB.^139^ The authoritative State Guide to Chemotherapy favoured a ‘preferred regime for the initial intensive … period of some 4–6 months’ of streptomycin and INH plus either ethambutol or ethionamide for children and pyrazinamide for adults.^140^ Through the late 1970s, the MOH repeatedly noted how limited was the use of rifampicin, due both to under-resourcing and the lack of short-course trial results.^141^ Thus, Cape Town and South Africa took a ‘conservative approach’, and only in 1980 was rifampicin's adoption in short-course therapy reported in the city.^142^

By the end of the 1970s the lack of improvement in notification rates was abundantly clear. An early survey by state epidemiologist H.G.V. Kustner found that notifications for tuberculous meningitis, ‘an index of the overall tuberculosis situation’, had not moved decisively up or down.^143^ In Cape Town, Black and Coloured peoples’ notifications had worsened (fig. 3) and MOH reports regularly invoked the ‘malnutrition, overcrowding, ignorance, cultural apathy and general socio-economic deprivation’ in explanation.^144^ AMR was now clearly linked to poorly managed chemotherapies, but, absent social improvement, the future seemed not to lie in antimicrobial restraint but in better, shorter treatment regimes with new drug combinations.

## Failures of prevention and cure: the National Control Programme under late apartheid, 1979–1989

The creeping sense of failure continued as sanctions against apartheid began to bite, underpinning negative epidemiological trends and health system pressures. South Africa's impressive economic growth rates of the 1960s (6% p.a.) and 1970s (3%) now fell to 1.2% in 1980–1987.^145^ Capital disinvestment and stalled employment followed, exacerbated by further American and European sanctions.^146^ Initially the health system experienced reductions of international aid, then real stresses on TB services began.^147^ Meanwhile, the HIV/AIDS pandemic returned TB to global prominence, albeit that the association of HIV with TB only slowly became apparent. New panaceas emerged, first INH prophylactic therapy, then supervised short-course combination treatment, later to segue into DOTS.^148^ Also influential was the WHO/UNICEF Alma-Ata initiative, championing ‘health for all’ through universal primary health care and reinforcing faith in ambulatory care.^149^ These themes appeared in South Africa's National Control Programme, which from 1980 prioritised community engagement and education, supervised short-course therapy, ‘passive’ case-finding through self-referrals and bacteriology, and BCG vaccination.^150^

How did this play out locally? Cape Town MOH reports in the early 1980s were already despairing. Pulmonary TB remained ‘the biggest public health problem … no significant progress has been made’. A defaulter rate of 30% on chemotherapy regimens was discovered, and rifampicin's promise of effective short-term treatment was nullified when the State Health Department ‘severely curtailed’ financial support.^151^ From late 1982, the City Council ceased providing rifampicin to new adult patients due to ‘insufficient funds’, and existing recipients received only low doses for 100-day stints; the Cape Divisional Council also truncated rifampicin regimens to under 6 months.^152^ Local officials’ frustration grew as South African evidence hardened for rifampicin's efficacy in combination regimens and for its net cost-effectiveness in short-course therapies.^153^ The results were soon evident. By 1984, Western Cape had the nation's highest TB incidence: 413 per 100,000 against a mean of c. 200.^154^ Sampling in 15 locations by the TBRI found that Coloured people here were the only group for whom prevalence and infection rates were not falling.^155^ Tuberculous meningitis incidence, the tracer for overall success, was particularly prominent in Cape Town, with case numbers highest amongst Coloured people and, ‘despite the use of BCG’, was ‘still distressingly common’ amongst Coloured children.^156^

In this situation the issue of ‘compliance’ loomed larger. An MOH survey of Cape Town clinic records up to 1982 concluded that 33% of TB patients were non-compliant, in that their attendance rates for treatment were below the minimum 75%.^157^ This, though, understated the true scale, for defaulters, out-migrants and the dead were excluded.^158^ Soon after, Cape Town researchers found that only half their sampled Black and Coloured TB clinic patients had compliance rates of 75% or more; 23% had rates below this, and 27% (tellingly) had no record.^159^ As to causes, they suggested poor ‘patient-therapist interaction’.^160^ A Cape Divisional Council study in 1981 followed 124 outpatients on short-course treatment in supervised settings, with 16.8% of the cohort defaulting before the end and a mean non-compliance rate of 88%. The relapse rate after 21 months, including defaulters, was 13.6%.^161^ Apartheid constraints prevented extensive research into reasons for default or non-compliance, but investigators suspected mobility due to migrant labour and residential segregation; patient self-diagnosis of recovery; distance of travel to clinic; preference for traditional healing; ignorance of TB's infectiousness and seriousness; and incompetent, lax, or indifferent professional oversight.^162^

All this mattered for AMR. The level of relapse uncovered in 1981 did not shake convictions that extra-short courses were basically effective, if only compliance could be addressed.^163^ This temptation towards complacency was exacerbated by clinical reports of defaulters from short-course treatments who resumed after a hiatus and achieved stable cures at follow-up.^164^ Meanwhile, resistance trend data was ambiguous. Cape Town's Regional Pathology Laboratory reported an average resistance level in sampled patients of 19% (1980–1985), falling to 15% by 1986, of which about half were monoresistant, with the remainder either two- or three-drug resistant.^165^ Nationally, INH primary resistance in the mid-1980s was found to be at 15% and acquired resistance at 50%.^166^ However, the TBRI drug surveillance sample suggested ongoing diminution of the problem. Resistance to INH fell from 23.2% (1971–1979) to 14.2% (1980–1988); rifampicin, 2% to 1.8%; ethionamide, 6.1% to 2.5%; ethambutol, 1.5% to 1.25%; with only streptomycin resistance bucking the downward trend, rising 10.6% to 12.1%.^167^ Optimistic doctors could reasonably assume AMR remained manageable.

In any event, alarm at AMR risks was increasingly eclipsed by other concerns. A partial state of emergency was imposed in 1985 following township protests that continued intermittently until apartheid ended. Cape Town was ‘racked with violent unrest’ which rendered ‘for long periods Guguletu and Langa … dangerous no-go areas’.^168^ Once again TB clinics and personnel were targets, with the Langa X-ray destroyed by arson, staff struggling to see patients, and the service ‘greatly hampered’. These challenges stretched a health system already under such resource constraint that the MOH declared: ‘We just cannot cope with this epidemic…’.^169^

Nationally, the frozen hospital building programme meant that all available TB beds were full.^170^ Loss of accommodation in the Cape was dire: South Africa's TB bed/patient ratio was 1:5, Cape Town's 1:25. Average stays shrank to less than 2 months rather than to the regimen's end.^171^ Thus, when TB officers warned of ‘the spectre of … the emergence of strains of drug resistant bacilli’, this was yoked to an argument about inadequate ambulatory care leading to non-compliance.^172^ AMR, then, was invoked as a rhetorical device to leverage funding for bed capacity and rifampicin supplies: ‘We are being cent wise, rand foolish and storing up a lot of trouble for the future’.^173^ By 1989, workload in badly underfunded clinics escalated; case-finding capacity dwindled as the Nyanga X-ray van deteriorated, notifications of Black and Coloured persons rose, and staffing to motivate poor compliers and trace defaulters was insufficient.^174^ TB, the MOH explained as he signed off, ‘is a socioeconomic disease’ for which medicines ‘will only have a short-term benefit’, albeit ‘… with our limited resources we are not even able to realise this’. Looking ahead, ‘it is not expected that the TB problem of the Cape Peninsula will decrease until the underlying problems that caused it are solved…’.^175^

## Social medicine, race, and modernity

This final *cri de couer* is a reminder that structural determinants of TB were never far from the policy discourse. Cape Town health officials alluded in the 1950s to ‘the social stresses and economic difficulties basically responsible for tuberculosis’; in the 1960s to ‘the socio-economic stresses militating against the non-European’ consequent on ‘the upsurge of industrialisation’; and in the 1980s to the ‘overcrowding and inadequate housing, unemployment, malnutrition, ignorance and apathy, and alcohol abuse’ that underlay infection.^176^

So too did the country's leading experts. Thus, Dormer in 1948 used ‘social diagnosis’ of a single case report to condemn:

‘Industry, economic need, Western civilisation…it is today's social system which was responsible for the death of our patient, as it is for the death-rate from tuberculosis of … industrial natives in South Africa today…’^177^

His solution was for industry to provide ‘proper housing … adequate food … recreation … pure water’, and state benefits to relieve tuberculous breadwinners of ‘prolonged mental strain and worry’.^178^ T.F.B. Collins was more circumspect in 1972, arguing that pharmaceuticalisation alone could not conquer ‘a multi-factoral problem’ turning on ‘socio-economic and environmental circumstances’ and requiring preventive measures like ‘practical encouragement to proper nutrition’ and ‘maintenance of good hygienic standards’.^179^ Likewise, in 1982, E. Glatthaar implicated the ‘socio-economic/nutritional problems in this country’ in causing thousands of ‘endogenous reactivations from the infected pool annually’; his solution was a combined state/SANTA initiative with community educators to address the ‘improvement of socio-economic and other stress conditions’.^180^

These allusions to structural factors evince changing assumptions about race. The mid-century sources capture the idealism of the Gluckman era, but their framing within racial hierarchies lent a paternalistic tone. Dormer's case report, for example, while rejecting arguments about innate susceptibility, depicted his African patient as a victim, in transition from ‘young warrior’ to degenerate shanty town dweller.^181^ Later writers emphasised behaviour, with the invocation of tropes like ‘apathy’ and ‘superstition’. Africans would be ushered towards modernity, with respect both to dietary choices that would strengthen immunity and to healthcare seeking that would catch TB early. Thus, upstream solutions gave way to individual responsibility.

We can view shifting approaches to social medicine through the prism of malnutrition. Alongside Gluckman's idealism, the 1940s saw a National Nutrition Council advocating free school meals, subsidies and redistribution of food surpluses.^182^ This Council, along with school feeding, was abolished by the NP on the grounds that taxpayers should not be funding welfare dependency.^183^ Medical lobbying for re-establishment led nowhere, and henceforth support came only from the third sector.^184^ In the Cape, the voluntary ‘Care Committee for Tuberculosis Patients’ focused on financial aid for rent and maintenance, providing clothing and blankets and, with SANTA, crèches for children of hospitalised patients; local government financed only almoner costs.^185^ The sole food aid came from the Peninsula School Feeding Association (PFSA), established in 1958 after government sustenance was withdrawn.^186^ Local lobbying for a provincial grant for the PFSA to support it ‘in combating Public Health Enemy No. 1, tuberculosis’ was unsuccessful.^187^ A traditional charity, operating street and cinema collections, auctions and individual fundraising, the PFSA dispensed bread, jam, peanut butter, ‘hot thick broth’ and skimmed milk for children in Langa, Gugulethu, and Nyanga.^188^ PSFA rhetoric was overtly oppositional: ‘Nearly every civilised country in the world including Soviet Russia has some form of school feeding for its future citizens’. Indeed, in 1963, its organising secretary, Jan Hoogendyk, was under a banning order and couldn't oversee fundraising.^189^ An Afrikaner anti-apartheid activist and member of the Congress of Democrats, Hoogendyk had been accused and acquitted at the Treason Trial and was later a political exile.^190^

Nutrition research also contained radical critique. In 1942, John Brock published the Cape Nutrition Survey, showing 49% of Coloured children were malnourished, against 37% European and 32% ‘Bantu’.^191^ Brock, who’d worked with British social medicine doyen John Ryle, blamed this on low incomes. A global expert in nutrition, he would later be ejected from South Africa's MRC for critical political comments.^192^ Attention later moved to protein malnutrition, with a Cape childrens’ hospital survey finding that 2% White, 8% Coloured, and 11% ‘Bantu’ were malnourished, some with kwashiorkor.^193^ That disease became notifiable in 1962, rescinded in 1968 due to the state's embarrassment at the scale uncovered.^194^ Under Grand Apartheid, racialised behavioural analyses became more common. After determining that White Cape schoolchildren were taller and heavier, researchers concluded that inter-racial differences in fitness were matters of genes, ‘training, skill and motivation’.^195^ Similarly a sugar industry-funded study of Black people's diets in Gugulethu combined empirical survey with racially determined assumptions, discussing food ‘taboos’, ‘pagan’ attitudes, ‘tribal differences’, ‘patent cures’ (including ‘asparagus for tuberculosis’), and ‘archetypal predeliction’ for carbohydrates.^196^

In the 1980s, evidence for structural inequalities became more insistent. A prospective cohort study of Cape Coloured children found 14% whose low birth weights indicated malnutrition due to poor maternal education, low income, and overcrowding.^197^ Black township children had similar levels of acute malnutrition, and anthopometric data showed a further 47% had nutritional stunting.^198^ Research lobbying for food supplementation reappeared in a study of cost-effectiveness of nutritional support in Cape Town's Crossroads squatter camp.^199^ Meanwhile, TB officials despaired of ever seeing structural interventions. ‘It is not possible to eradicate tuberculosis by medical means alone. The public health worker can but reiterate these unpleasant truths time after time to successions of policy makers’.^200^

## Conclusion

Writing in 2000, the physician-anthropologist Paul Farmer argued for ‘pragmatic solidarity’ in the care of TB patients.^201^ By this he meant that it was inappropriate to blame the sick for failures of treatment regimes. Rather, doctors ought to adopt a person-centred approach, adjusting the system to facilitate access, ever-mindful of the poverty, and structural constraints facing their patients. The challenge of care, then, is to improve the social bonds between doctor and patient at the ‘threshold where social and biological aspects of disease are negotiated’ and so to improve adherence to therapies’.^202^ In thinking about ameliorating these threshold relationships today, it is instructive to reflect on the history reviewed here, which provides an extreme example of the limits of TB medicine in a setting of structural violence.

The aim of the study was to examine in depth how, at the level of public health, TB was managed in an apartheid society. Our closing argument, from the mouths of TB doctors themselves, is a familiar one: failure to address nutrition, housing, incomes and work undermined prevention. Social medicine was always peripheral in a country which, outside the circle of White settler privilege, considered itself ‘developing’. South Africa's far right governments, hostile to redistribution and pro-private medicine, could never countenance structural solutions. Pharmaceuticalisation provided the cover, but these were never seriously on the table.

Beyond that, what have we shown? The Cape's chronology varies somewhat from the narrative of TB historiography. BCG programmes became significant only gradually and seemed less central to overall policy, which prioritised screening and drug treatment. The fatal entanglement of disease surveillance with population policing bred antipathy and encouraged periodic attacks on health facilities. Ambulatory care emerged early and by necessity, for hospital capacity was insufficient. Choice of drug regimes appears fairly consistent through the period, with rifampicin too expensive and scarce to become the much-heralded panacea. These were the circumstances in which awareness of AMR, and relatedly combination therapies, first emerged in the 1950s and 1960s. Problems of ‘defaulting’ and ‘compliance’ were well understood by the 1970s, even if no systematic enquiry to inform policy response was undertaken. Monitoring of primary and acquired mono- and multi-drug resistance was underway from the late 1960s, although an apparent downward trend in rates by the 1980s may have instilled false security. The policy priority was therefore not the preservation of antibiotic efficacy, but rather the more effective management of treatment. Hence, the country was poorly prepared for the HIV/AIDS-driven rise in incidence at the end of our period.

We cannot meaningfully calibrate the extent to which apartheid worsened this situation relative to other countries whose polities were not premised on White supremacy. However, our discussion adds further weight to the ‘failure’ literature. There were direct impacts: sanctions constraining resources, and the destruction of facilities. There was the cultural gulf of presumption and resentment which vitiated policies of health education and community engagement. There was the hopelessness of monitoring and managing populations whose circumstances and motivations were shaped by residential segregation, labour mobility and state intrusion. Above all, there was the impossibility, in a society premised on racial difference and restricted civil rights, of imagining and treating TB as a universal problem.

## Footnotes

1 R. and J. Dubos, *The White Plague: Tuberculosis, Man, and Society*, 1987 edn (New Brunswick: Rutgers University Press, 1952), 207.

2 Paul Farmer, ‘The Consumption of the Poor’, in Haun Saussy, ed, *Partner to the Poor A Paul Farmer Reader* (London: University of California Press, 2010), 222–47, at 223.

3 R. and J. Dubos, *White Plague,* 207; Paul Farmer, ‘Social Scientists and the New Tuberculosis’, in Saussy ed., *Partner to the Poor,* 174–94, at 177.

4 A. McDowell, *Breathless: Tuberculosis, Inequality, and Care in Rural India* (Stanford: Stanford University Press, 2024), 9–10.

5 McDowell, *Breathless,* 10–11; Janina Kehr, ‘‘It's also the system’ Republican Dilemmas in French Tuberculosis Prevention’, in Helen Macdonald and Ian Harper, eds, *Understanding Tuberculosis and its Control* (London: Routledge, 2019), 88–105.

6 Elizabeth L. Corbett et al., ‘Tuberculosis in Sub-Saharan Africa: Opportunities, Challenges, and Change in the Era of Antiretroviral Treatment’, *Lancet,* 367 (2006), 926–37.

7 ‘State of the Union's Health: Minister's Review’, *South African Medical Journal (SAMJ)*, 32 (1958), 875–76, at 876; P. B. Fourie et al., ‘Current Views on Tuberculosis’, *South African Journal of Science (SAJS)*, 82 (1986), 386–95 at 386; Statistics South Africa, *Mortality and Causes of Death in South Africa: Findings from Death Notification 2020* (Pretoria: Stat sa), 40–43.

8 A. M. Beemer and B. A. Dormer, ‘Bacterial Sensitivity to Anti-Microbial Agents. A Warning Against Wanton Waste of Antibiotics and Chemotherapeutic Substances’, *SAMJ*, 26 (1952), 311–13 at 311; J. B. Porteous, ‘The Treatment of Pulmonary Tuberculosis’, *SAMJ*, 33 (1959), 13, 265–67, 266.

9 Christian W. McMillen, *Discovering Tuberculosis: A Global History, 1900 to Present* (New Haven: Yale, 2015), 224–29; Bharat Jayram Venkat, *At the Limits of Cure* (Durham: Duke University Press, 2021).

10 N. Brimnes, *Languished Hopes: Tuberculosis, the State and International Assistance in Twentieth-Century India* (New Delhi: Orient Longman, 2016).

11 McMillen, *Discovering Tuberculosis*, 37–56; M. Worboys, ‘Tuberculosis and Race in Britain and its Empire, 1900–1950’, in W. Ernst and B. Harris, *Race, Science and Medicine* (London: Routledge, 1999), 144–66.

12 Niels Brimnes, ‘Rallying around the Magic Wand: Visions of Social Medicine, Public Health and Disease Control in India 1946–1957’, in Iris Borowy and Bernard Harris, eds, *Health and Development* (München: De Gruyter Oldenbourg, 2023), 175–99.

13 Sunil Amrith, *Decolonizing International Health: India and Southeast Asia 1930–65* (Basingstoke: Palgrave, 2006), 57–63.

14 M. C. Raviglione, A. Pio, ‘Evolution of WHO Policies for Tuberculosis Control, 1948–2001, *Lancet*, 35 (2002), 775–80.

15 Niels Brimnes, ‘BCG Vaccination and WHO's Global Strategy for Tuberculosis Control 1948–1983’, *Social Science & Medicine*, 67 (2008), 863–73; Brimnes, *Languished Hopes*.

16 McMillen, *Discovering Tuberculosis*, 71–90.

17 L. Monnais, *The Colonial Life of Pharmaceuticals: Medicines and Modernity in Vietnam* (London: Cambridge University Press, 2019).

18 Christoph Gradmann, ‘This Is the End: Eradicating Tuberculosis in Modern Times’, *Centaurus*, 64 (2022), 171–80, at 176; McMillen, *Discovering Tuberculosis*, 119–67.

19 Christoph Gradmann, ‘Magic Bullets and Moving Targets: Antibiotic Resistance and Experimental Chemotherapy, 1900–1940’, *Dynamis*, 31 (2011), 305–21; Scott H. Podolsky, *The Antibiotic Era: Reform, Resistance, and the Pursuit of a Rational Therapeutics* (Baltimore: Johns Hopkins University Press, 2015), 140–79.

20 Christoph Gradmann, ‘Treatment on Trial: Tanzania's National Tuberculosis Program, the International Union against Tuberculosis and Lung Disease, and the Road to DOTS, 1977–1991’, *Journal of the History of Medicine and Allied Sciences*, 74 (2019), 316–43; McMillen, *Discovering Tuberculosis*, 203–209.

21 Barron H. Lerner, ‘From Careless Consumptives to Recalcitrant Patients: The Historical Construction of Noncompliance’, *Social Science & Medicine*, 45 (1997), 1423–31; McMillen, *Discovering Tuberculosis*, 149–65; Jens Seeberg, ‘Biosocial Dynamics of Multidrug-Resistant Tuberculosis’, in Jens Seeberg, Andreas Roepstorff, and Lotte Meinert, eds, *Biosocial Worlds: Anthropology of Health Environments Beyond Determinism* (London: UCL Press, 2020), 124–45, 138.

22 McMillen, *Discovering Tuberculosis*, 43.

23 Dingie Van Rensburg et al., ‘Tuberculosis Control in South Africa: Reasons for Persistent Failure’, *Acta Academica* 1 (2005), 1–55, at 42.

24 E. Glatthaar, ‘Tuberculosis Control in South Africa: Where Have We Gone Wrong? and a Look into the Future’, *SAMJ* 62 (1982), 36–41; T. F. B. Collins, ‘The History of Southern Africa's First Tuberculosis Epidemic, *SAMJ,* 62 (1982), 780–88.

25 Randall Packard, *White Plague, Black Labor: Tuberculosis and the Political Economy of Health and Disease in South Africa* (Los Angeles: University of California Press, 1989).

26 Packard, *White Plague,* 249–52, 292–98.

27 Jock McCulloch and Pavla Miller, *Mining Gold and Manufacturing Ignorance. Occupational Lung Disease and the Buying and Selling of Labour in Southern Africa* (Singapore: Palgrave, 2023).

28 Van Rensburg et al., ‘Tuberculosis Control in South Africa’, 19–25.

29 Van Rensburg et al., ‘Tuberculosis Control in South Africa’, 42.

30 Elizabeth van Heyningen, ‘Epidemics and Disease: Historical Writing on Health in South Africa’, *South African Historical Journal,* 23 (1990), 122–33.

31 Valerie Zangel, ‘Pulmonary Tuberculosis in Cape Town and the Karoo, 1870–1920: Policy and Attitudes’ (unpublished PhD thesis, University of South Africa, 2017).

32 Zangel, ‘Pulmonary Tuberculosis in Cape Town’, 2017, 126–8, 131–59, 238–42; N. Ndjeka et al., ‘High Treatment Success Rate for Multidrug-Resistant and Extensively Drug-Resistant Tuberculosis Using a Bedaquiline-Containing Treatment Regimen’, *European Respiratory Journal,* 52 (2018).

33 M. Adhikari, *Not White Enough, Not Black Enough—Racial Identity in the South African Coloured Community* (Cape Town: Double Storey, 2006); Z. Erasmus, *Coloured by History, Shaped by Place: New Perspectives on Coloured Identities in Cape Town* (Cape Town, Maroelana: Kwela Books, 2001); E. Cavanaugh, *The Griqua Past and the Limits of South African History, 1902–1994* (Oxford: Peter Lang, 2011).

34 M. Mbali, ‘“A Matter of Conscience”: The Moral Authority of the World Medical Association and the Readmission of the South Africans, 1976–1994’, *Medical History*, 58 (2014), 257–77; ‘MASA to End Affiliation with BMA’, *BMJ,* 284 (1982), 1971; J. de Gruchy et al., ‘The Difficult Road to Truth and Reconciliation–The Health Sector Takes its First Steps’, *SAMJ*, 88 (1998), 975–79.

35 Deborah Posel, ‘What's in a Name? Racial Categorisations under Apartheid and Their Afterlife’, *Transformation*, 47 (2001), 50–74; Deborah Posel, ‘Race as Common Sense: Racial Classification in Twentieth-Century South Africa’, *African Studies Review,* 44 (2001), 87–114.

36 Ann Laura Stoler, ‘Colonial Archives and the Arts of Governance’, *Archival Science*, 2 (2002), 87–109; Antoinette Burton, ed, *Facts, Fictions, and the Writing of History* (Durham, NC: Duke University Press, 2005).

37 Yann Blouin et al., ‘Significance of the Identification in the Horn of Africa of an Exceptionally Deep Branching *Mycobacterium tuberculosis* Clade’, *PLoS ONE*, 7 (2012), e52841; C. Metcalf, ‘A History of Tuberculosis’, in H. M. Coovadia and S. R. Benatar, eds, *A Century of Tuberculosis: South African Perspectives* (Cape Town: Oxford University Press, 1991), 1–28, 21.

38 Zangel, ‘Pulmonary Tuberculosis in Cape Town’, 32–47.

39 R. Packard, ‘Holding Back the Tide: TB Control Efforts in South Africa’, in Coovadia and Benatar, eds, *Century of Tuberculosis*, 42–56.

40 Susan Parnell, ‘Creating Racial Privilege: The Origins of South African Public Health and Town Planning Legislation’, *Journal of Southern African Studies*, 19 (1993), 471–88.

41 L. Fatti et al., ‘Pulmonary Tuberculosis: The Results of Treatment at Baragwanath Non-European Hospital’, *SAMJ*, 24 (1950), 1065–69; Fiona Kilpatrick, ‘Consumptive Cape Town: the Chapel Street TB Clinic, 1941–1964’ (unpublished MA thesis, University of Cape Town, 2002), 103.

42 Cape Town City Health Dept, *Annual Report of the Medical Officer of Health [to] the Corporation of the City of Cape Town* Cape Times (hereafter MOH AR) 1948, 45; Kilpatrick, ‘Consumptive Cape Town’, 59, 62.

43 Kilpatrick, ‘Consumptive Cape Town’, 64–67, 84–86.

44 Western Cape Archives and Records Service (WCARS), ‘Control and Administration of Langa Native Hospital’ 11 Feb 1950, City Engineer's Department Correspondence, 14/5/6; ‘Increased Hospital Accommodation at Cape Town’, *South African Medical Record*, 22 September 1947, 429.

45 Nigel Worden, *The Making of Modern South Africa: Conquest, Segregation, and Apartheid* (Cambridge: Blackwell, 1995), 61; City of Cape Town Housing Committee (23 December, 1958), 1133.

46 Kilpatrick, ‘Consumptive Cape Town’, 72–82.

47 Union of South Africa, ‘Group Areas Act’, Pub. L. No. 41 (1950).

48 Geoffrey Bowker and Susan Star, *Sorting Things Out: Classification and Its Consequences* (Cambridge: MIT Press, 1999), 195–225; Worden, *Making of Modern South Africa*, 61, 96–97.

49 Uma Shashikant Mesthrie, ‘“No Place in the World to go to” Control by Permit: The First Phase of the Group Areas Act in Cape Town in the 1950s’, in Elizabeth van Heyningen, ed, *Studies in the History of Cape Town Volume 7* (Rondebosch: UCT Press, 1994), 184–207.

50 H. Gluckman, ‘National Health Service: Address by Dr. The Honourable Henry Gluckman, Minister of Health’, *SAMJ* 20 (1946), 655–63; H. T. Phillips, ‘The 1945 Gluckman Report and the Establishment of South Africa's Health Centers’, *American Journal of Public Health,* 83 (1993), 1037–38.

51 Shula Marks, ‘South Africa's Early Experiment in Social Medicine: Its Pioneers and Politics’, *American Journal of Public Health*, 87 (1997), 3.

52 Gluckman, ‘National Health Service’.

53 National Health Services Commission, *Report of the National Health Services Commission on the Provision of an Organized National Health Service for All Sections of the People of the Union of South Africa, 1942–1944* (Pretoria: Government Printer, 1944), 25–31, 44, 76–77, 98, 145, 177.

54 J. F. Brock and M. Rollo, ‘The Cape Coloured People: their Pattern of Health and Disease’, *SAMJ*, (1949), 1000–10, 1008–1009.

55 Marks, ‘South Africa's Early Experiment’.

56 Hoosen Coovadia et al., ‘The Health and Health System of South Africa: Historical Roots of Current Public Health Challenges’, *Lancet*, 374 (2009), 817–34.

57 MOH AR 1949.

58 MOH AR 1949, 41–42.

59 MOH AR 1948, 46.

60 MOH AR 1951, 42; Stals was the NP's first Minister of Health.

61 Anon, ‘The DP Marais SANTA Centre at Westlake’, *SAMJ* 31 (1957), 732–33.

62 T. Collins, ‘The Birth of SANTA: A Short History of the Voluntary Organisations in the Fight against Tuberculosis in South Africa’, *SANTA News*, (1987), 3–8; ‘Editorial: SANTA at Work for Three Decades’, *SANTA Bantu Magazine*, May 1, 1977; Letter to Director of Medical Services, ‘Plans for the Establishment of Tuberculosis Settlements throughout the Union’, *Cape Town Archives* (1952).

63 ‘DP Marais SANTA Centre’, 733; Cape Divisional Council covered the city's rural hinterland, Stellenbosch and Paarl.

64 MOH AR 1955, 45, 51.

65 MOH AR, 1963, 52; MOH AR, 1967, 2; Kilpatrick, ‘Consumptive Cape Town’, 105–106.

66 MOH AR, 1973; MOH AR 1978, 2, Table v.17, 148; MOH AR, 1984, 2.

67 M. Minde, ‘History of Mental Health Services in South Africa—Part X. Institutions for Defectives’, *SAMJ*, 49, 1890–94; MOH AR, 1984, vi, 84.

68 Neil Anderson and Shula Marks, ‘Apartheid and Health in the 1980s’, *Social Science and Medicine*, 27 (1988), 667–81, 678.

69 Max Price, ‘The Impact of Political Transformation in South Africa on Public/Private Mix Policy Debates’, *Health Policy and Planning*, 9 (1994), 50–62, 52.

70 Elizabeth Thomson, ‘The Private Hospital Industry in the Greater Cape Town area’, *SAMJ*, 66 (1984), 17–20.

71 Price, ‘Impact of Political Transformation’, 52–55.

72 Thomson, ‘Private Hospital Industry’, 18–19.

73 MOH AR 1950, 47–48.

74 C. Sheldon et al., ‘Notification of Tuberculosis: How Many Cases are Never Reported?’, *Thorax*, 47 (1992), 1015–18; D. Wilkinson et al., ‘Tuberculosis Treatment Programmes in Low-Income Countries’, *Lancet*, 344 (1994), 259–60.

75 MOH AR 1961, 12.

76 Policy and Strategy Department, *Census 2022: Cape Town Trends and Changes* (City of Cape Town, 2023), 3.

77 MOH AR 1948, 39; 1959, 48; 1963, 55.

78 MOH AR 1989, 37.

79 MOH AR 1951, 39–40.

80 MOH AR 1949, 40.

81 WCARS, ‘Correspondence: MOH to Chairman of Health Comm—Tuberculosis—Langa Location (11 Sep 1958)’, September 11, 1958.

82 MOH AR 1951, 40; 1955, 42; 1958, 4, 48.

83 MOH AR 1960, 5, 48–49.

84 MOH AR 1961 52; 1964, 53.

85 MOH AR 1967, 2, 54; 1971, 60.

86 MOH AR 1977, 69.

87 MOH AR 1977, 61; 1987, 64.

88 MOH AR 1981, 2, 74; 1984, 79–80; 1987, i, 64.

89 E. M. Thomson and S. Myrdal, ‘Regional Variations in Tuberculosis Policy in the Cape and Ciskei’, *SAMJ*, 70 (1986), 253–57, 253.

90 WCARS, ‘Addendum in Letter: MOH to Chairman and Members of Health Committee—Tuberculosis: Screening of Pupils at Schools (1 May 1964).’

91 Brimnes, ‘BCG Vaccination’; McMillen, *Discovering Tuberculosis*, 84–118.

92 B. A. Dormer, ‘Control of Tuberculosis: The Role of BCG and Vole Vaccines’, *SAMJ*, 23 (1949), 488–90.

93 J. Arthur Myers, ‘The Ever-Continuing Search for Immunity in Tuberculosis’, *SAMJ*, 27 (1953), 169–71, 171.

94 Anon, ‘Vaccination Against Tuberculosis, *SAMJ*, 28 (1954), 964; Anon, ‘BCG and Vole-Vacillus Vaccines in the Prevention of Tuberculosis in Adolescents, *SAMJ*, 30 (1956), 388–91; J. P. de Villiers, ‘The Tuberculosis Problem in South Africa in the Light of Recent Advances’, 31 (1957), 1274–77; MOH AR 1959, 5, 25, 32.

95 MOH AR 1963, 37.

96 MOH AR 1964, 57.

97 T. F. B. Collins and A. Broomberg, ‘Prevention of Tuberculosis—BCG in Diagnosis and Prophylaxis’, *SAMJ,* 39 (1965), 89–92.

98 M. Spencer et al., ‘A Field Experience of Mass Percutaneous BCG Inoculation as an Immunizing and Diagnostic Procedure’, *SAMJ*, 38 (1964), 223–33.

99 MOH AR 1978, 43.

100 T. F. B. Collins, ‘Limitations of the Accelerated BCG Reaction as a Test for Previous Infection with the Tubercle Bacillus’, *SAMJ,* 39 (1965), 281–82.

101 J. Geefhuysen and I. Freiman, ‘Tuberculosis notwithstanding BCG vaccination’, *SAMJ*, 49 (1975), 1706–708.

102 W. J. Ijsselmuiden et al., ‘Notification of Five of the EPI Target Diseases in South Africa—An Assessment of Disease and Vaccination Reporting’, *SAMJ,* 72 (1987), 311–16, 315.

103 P. B. Fourie, ‘BCG Vaccination and the EPI’, *SAMJ,* 72 (1987), 323–26, 323.

104 MOH AR 1987, 50; MOH AR WC 1989, 46; Thomson and Myrdal, ‘Regional Variations’, 256.

105 B. A. Dormer, ‘Tuberculosis in South Africa’, *Journal of the South African Veterinary Association*, 28 (1957), 195–204, 197.

106 MOH AR 1948, 44; MOH AR 1951, 41.

107 MOH AR 1951, 42.

108 WCARS, ‘Outpatient Services: Langa Native Hospital’, July 13, 1956.

109 E. D. Cooper, ‘The Domiciliary Treatment of Pulmonary Tuberculosis as a Public Service’, *SAMJ*, 28 (1954), 197.

110 Collins, ‘First Tuberculosis Epidemic’, 786.

111 Kilpatrick, ‘Consumptive Cape Town’, 127–30.

112 MOH AR 1963, 4.

113 MOH AR 1955, 4, 41, 44.

114 de Villiers, ‘Tuberculosis Problem’, 1275.

115 MOH AR 1958, 53.

116 de Villiers, ‘Tuberculosis Problem’, 1277.

117 MOH AR 1958 48–50.

118 MOH AR 1959, 45; 1961, 56.

119 WCARS, ‘Report: Provision of Mass X-Ray Unit for Bantu Work-Seekers: City of Cape Town (28 Sep 1966)’, Mass X-Ray Unit Bantu Work Seekers 3/CT (1966).

120 MOH AR 1967, 2.

121 WCARS, ‘Letter: The Secretary (Lighton) to Town Clerk—Tuberculosis (9 Jun 1959)’; ‘Mobile Clinic for TB Sifting’, Cape Times, February 27, 1958; ‘Letter: MoH to Town Clerk—Mobile Clinic and Combatting of TB (14 Jan 1958)’; ‘Letter: MoH to Town Clerk—Tuberculosis (1 Jul 1959)’; ‘Letter: Town Clerk to The Secretary (Lighton) Cape Chamber of Industries—X-Ray Facilities (10 Feb 1959)’.

122 WCARS, ‘Assistance to Indigent TB Sufferers and Their Dependants’ Vol: 4/1/1, no. 3/CT Ref: 2/5/1 (1959).

123 MOH AR 1959, 49.

124 B. A. Dormer, ‘Tuberculosis’, *SAMJ*, 34 (1960), 291–93, 293.

125 MOH AR 1958, 53.

126 A. D. Ginsburg et al., ‘Resistant Tuberculosis in an Inpatient Hospital Population in Cape Town’, *SAMJ*, 41 (1967), 1162–66.

127 T. F. B. Collins, ‘The New Approach to Tuberculosis’, *SAMJ*, 46 (1972), 260–61.

128 T. F. B. Collins, ‘Tuberculosis Control in An Urban Area’, *SAMJ,* 42 (1968), 1012–14; see also J. De Beer, ‘A Forward View of Health Services in South Africa’, *SAMJ,* 50 (1976), 431–40, 434–35.

129 MOH AR 1970 50; 1971, 51–52, 56.

130 MOH AR 1975, 58.

131 MOH AR 1972, 2, 56, 61.

132 MOH AR 1976 61; 1978 51.

133 MOH AR 1977 70.

134 Borthwick Institute for Archives: CSAS/CA 11.2 *Preliminary Chronology of Events in Cape Town in the Unrest Following the Soweto Riots.*

135 MOH AR 1970–1980 passim.

136 Glatthaar, ‘Tuberculosis Control’, 37.

137 K. Weyer and H. H. Kleeberg, ‘Primary and Acquired Drug Resistance in Adult Black Patients with Tuberculosis in South Africa: Results of a Continuous National Drug Resistance Surveillance Programme Involvement’, *Tubercle and Lung Disease*, 73 (1992), 106–12.

138 P. L. Salinger, ‘Evaluation of Tuberculosis Control Measures. The 2nd Basil Dormer Lecture, 1977’, *SAMJ,* 52 (1977), 1136–37; M. D. Holland, ‘Rifampicin—What Dose for Pulmonary Tuberculosis?’, *SAMJ*, 46 (1972), 604–608; J. Bell and D. Yach, ‘Tuberculosis Patient Compliance in the Western Cape, 1984,’ *SAMJ*, 73 (1988), 31–33; Collins, ‘New Approach to Tuberculosis’.

139 MOH AR 1975, 58, 63.

140 MOH AR 1978 54.

141 MOH AR 1978 54; MOH AR 1979 72.

142 MOH AR 1980 82–83.

143 H. G. V. Kustner, ‘Trends in Four Major Communicable Diseases’, *SAMJ*, 55 (1979), 460–73, 464.

144 MOH AR 1978, 49.

145 Roger C. Ridell, ‘The Impact of New Sanctions Against South Africa’, in Robert E. Edgar, ed, *Sanctioning Apartheid* (Trenton: Africa World Press, 1990), 111–39, 113–14.

146 Ridell, ‘Impact of New Sanctions’, 115–25.

147 Yach et al., ‘Use of Indicators’, 806.

148 McMillen, *Discovering Tuberculosis*, 186–201; Gradmann, ‘Treatment on Trial’.

149 Gradmann, ‘Treatment on Trial’, 330–31.

150 D. Yach et al., ‘Use of Indicators in Achieving ’Health for All' in South Africa, *SAMJ*, 72 (1987), 805–807; Glatthaar, ‘Tuberculosis Control’, 39; S. Hermans et al., ‘The Mass Miniature Chest Radiography Programme in Cape Town, South Africa, 1948–1994: The Impact of Active Tuberculosis Case Finding’, *SAMJ*, 106 (2016), 1263–69.

151 MOH AR 1981 4, 80.

152 Thomson and Myrdal, ‘Regional Variations’, 254.

153 S. D. Miller, ‘A Comparative Evaluation of Drug Combinations Used in the Treatment of Pulmonary Tuberculosis’, *SAMJ*, 63 (1983), 764–66; L. R. Tibbit, ‘The Economics of Short-Course Therapy for Tuberculosis in the Cape Divisional Council Area’, *SAMJ*, 62 (1982), 31–33.

154 H. G. V. Kustner, ‘Tuberculosis Notifications: An Up-date’, *SAJS*, 82 (1986), 386–87.

155 P. Fourie and Karin Knoetze, ‘Tuberculosis Prevalence and Risk of Infection in Southern Africa’, *SAJS*, 82 (1986), 387.

156 M. A. Kibel et al., ‘Tuberculosis Meningitis in the Western Cape from 1979–84: Epidemiology and Outcome’, *SAJS*, 82 (1986), 387–88.

157 MOH AR 1984, 83–84.

158 S. M. Youngleson, ‘Measuring Patient Compliance in the Treatment of Pulmonary Tuberculosis in Cape Town—Pitfalls in Study Design’, *SAMJ*, 73 (1988), 28–30.

159 Elizabeth M. Thomson and Susan Myrdal, ‘The Implementation of Tuberculosis Policy in Three Areas in South Africa’, *SAMJ,* 70 (1986), 258–62, 261.

160 Thomson and Myrdal, ‘Regional Variations’, 256.

161 S. A. Fisher, ‘Experiences with Extra-short course Tuberculosis Chemotherapy in the Divisonal Council of the Cape’, *SAJS*, 82 (1986), 393; the Council defined default as missing four or more consecutive treatments, or 11 in a month: Thomson and Myrdal, ‘Regional Variations’, 255.

162 M. Zabow and J. O. Pearson, ‘Short-course Chemotherapy for Pulmonary Tuberculosis—Points of Interest’, *SAMJ*, 61 (1982), 867–70; M. L. Griffiths et al., ‘Tuberculosis Management in a Rural Community—Factors in Failure’, *SAMJ,* 59 (1981), 14–16; E. M. Thomson and S. Myrdal, ‘Tuberculosis—the Patients’ Perspective’, *SAMJ,* 70 (1986), 263–64.

163 Fisher, ‘Extra-Short Course Tuberculosis Chemotherapy’, 393.

164 Zabow and Pearson, ‘Short-Course Chemotherapy’.

165 C. Byrnes and G. J. Coetzee, ‘Mycobacterium Tuberculosis Resistance in the Western Cape’, *SAJS*, 82 (1986), 394.

166 Fourie and Knoetze, ‘Tuberculosis Prevalence’, 387.

167 Weyer and Kleeberg, ‘Primary and Acquired Drug Resistance’, 109.

168 MOH AR 1985 i, iii.

169 MOH AR 1985 v, ix.

170 H. H. Kleeberg, ‘The Dynamics of Tuberculosis in South Africa and the Impact of the Control Programme’, *SAMJ*, 62 (1982), 22–23.

171 MOH AR 1984, vi, 84.

172 MOH AR 1984, vi.

173 MOH AR 1985, ix.

174 MOH AR 1989, 4, 31, 45–47.

175 MOH AR 1989, 47.

176 MOH AR 1951, 42; 1963, 51; 1978, 49; 1989, 47.

177 B. A. Dormer, ‘A Case of Tuberculosis’, *SAMJ*, 22 (1948), 82–88, at 85.

178 Dormer, ‘Case of Tuberculosis’, 87–88.

179 Collins, ‘New Approach’, 261.

180 Glatthaar, ‘Tuberculosis Control’, 37, 39.

181 Dormer, ‘Case of Tuberculosis’.

182 Diana Wylie, *Starving on a Full Stomach: Hunger and the Triumph of Cultural Racism in Modern South Africa* (Charlottesville: University of Virginia Press, 2001), 209–15.

183 Wylie, *Starving on a Full Stomach*, 216–19, 224.

184 J. P. de Wit, ‘The South African Nutrition Society and its role’, *SAMJ*, 49 (1975), 2211–14.

185 For example, MOH AR 1973, 60.

186 WCARS 3CT MOH Box 13. 70 ‘Feeding Scheme Indigent TB Out-patients 1957–60’, File 70 1958–1961 ‘Feeding of School Children’ Minutes of the Peninsula School Feeding Association (PSFA) 4/3/1963, 3/6/1963, 1/7/1963, 6/7/1964.

187 WCARS 3CT MOH Box 13. 70 ‘Memorandum on the Re-introduction of a Modified School Feeding Scheme’, April 29, 1959.

188 WCARS Minutes PFSA 3/6/1963, 1/7/1963, 6/7/1964.

189 WCARS Minutes PSFA 4/3/1963, 3/6/1963.

190 Jan Hoogendyk, *According to My Beliefs* (Banbury, Counterpoint, 1995).

191 J. F. Brock and J. M. Latsky, ‘The Findings of the Cape Nutrition Survey’, *SAMJ,* 16 (1942), 255–60.

192 Wylie, *Starving on a Full Stomach*, 149, 156–60, 224.

193 F. J. Ford et al., The Clientele of a Children's Hospital in Cape Town, *SAMJ*, 35 (1961), 1081–85.

194 Wylie, *Starving on a Full Stomach*, 222–24.

195 A. W. Sloan and J. D. L. Hansen, ‘Nutrition and Physical Fitness of White, Coloured and Bantu High-School Children’, *SAMJ*, 43 (1969), 508–11, 511.

196 E. B. Manning et al., ‘Dietary Patterns in Urbanised Blacks–A Study in Guguletu, Cape Town, 1971’, *SAMJ*, 48 (1974), 485–98.

197 J. P. G. Molteno et al., ‘A Study on Child Development in Cape Town—The Cohort and Sample’, *SAMJ*, 58 (1980), 729–32.

198 C. T. Hugo-Hamman et al., ‘Nutrition Status of Pre-School Children in a Cape Town Township’, *SAMJ*, 72 (1987), 353–55.

199 D. E. Whittaker et al., ‘The Cost Effectiveness of the Philani Nutrition Day Centre in Crossroads Squatter Camp, Cape Town, *SAMJ*, 68 (1985), 174–76.

200 MOH AR 1985, ix.

201 Farmer, ‘Consumption of the Poor’, 242–43.

202 Erin Koch, ‘Tuberculosis Is a Threshold: The Making of a Social Disease in Post-Soviet Georgia′, *Medical Anthropology*, 32 (2013), 309–32.

